# Vitamin D and Cardiovascular Disease

**DOI:** 10.3390/nu2040426

**Published:** 2010-03-31

**Authors:** Vivian Cristina Garcia, Lígia Araújo Martini

**Affiliations:** Nutrition Department, School of Public Health, Sao Paulo University, Av. Dr. Arnaldo, 715, Cerqueira César, CEP 01246-904, São Paulo, Brazil; Email: viviangarcia@usp.br

**Keywords:** vitamin D, cardiovascular disease, hypertension

## Abstract

Vitamin D insufficiency/deficiency has been observed worldwide at all stages of life. It has been characterized as a public health problem, since low concentrations of this vitamin have been linked to the pathogenesis of several chronic diseases. Several studies have suggested that vitamin D is involved in cardiovascular diseases and have provided evidence that it has a role in reducing cardiovascular disease risk. It may be involved in regulation of gene expression through the presence of vitamin D receptors in various cells, regulation of blood pressure (through renin-angiotensin system), and modulation of cell growth and proliferation including vascular smooth muscle cells and cardiomyocytes. Identifying correct mechanisms and relationships between vitamin D and such diseases could be important in relation to patient care and healthcare policies.

## 1. Introduction

The main function of vitamin D relates to the development and maintenance of bone tissue. It is responsible for maintaining calcium and phosphorus homeostasis. Vitamin D insufficiency/deficiency has been observed worldwide at all stages of life. It has been characterized as a public health problem, since low concentrations of this vitamin has been linked to the pathogenesis of several chronic diseases with cardiovascular risk factors, such as hypertension, heart failure, atherosclerosis and peripheral arterial disease [1,2,3]. Following the discovery of the presence of vitamin D receptors (VDR) in many cells, including cardiomyocytes [4], vascular smooth muscle cells (VSMC) [5] and endothelium [6], several mechanisms have been proposed to explain the relationship between vitamin D and the development of cardiovascular disease. Such mechanisms include involvement of vitamin D in the angiotensin-renin system [7] and proliferation and growth of VSMC [8]. 

## 2. Physiology of Vitamin D

Vitamin D is found as ergocalciferol (vitamin D_2_) produced by plant and as cholecalciferol (vitamin D_3_) produced by animal tissue. It is also produced by exposure to ultraviolet-B (290 to 310 nm) in 7-dehydrocholesterol, which is present in human skin [9]. It has been estimated that 80-90% of vitamin D is acquired by means of cutaneous synthesis and the remainder through the diet [10]. Vitamin D prohormone is biologically inactive, and it becomes active through conversion to its major form 25-hydroxyvitamin D (25(OH)D) in the liver: this metabolite is used to classify vitamin D status. Following this, the hormonal form of vitamin D (1,25-dihydroxyvitamin D [1,25(OH)2D3] or calcitriol) is produced in other tissues like prostate, breast, colon and especially the kidneys, through 1-alpha-hydroxylase [9,10]. This metabolite production is controlled by the serum parathyroid hormone (PTH), calcium and phosphorus concentrations. 

The effects of 1,25(OH)_2_D_3 _are mediated by VDR, which are present in many cells. At the nucleus of the target cells, 1,25(OH)_2_D_3 _associated with VDR binds to the retinoic X receptor (RXR), thereby forming heterodimers. These work on vitamin D response elements, hence initiating the cascade of molecular interactions that will modulate the transcription of the specific gene [11]. Thus, exceedingly low concentrations of 25(OH)D can result in failure of this metabolic cascade and alter gene expression.

Individuals’ vitamin D levels or their nutritional status regarding vitamin D are measured according to the plasma levels of 25(OH)D. The biologically active form of vitamin D, (1,25(OH)_2_D_3_), is unsuitable for this purpose for reasons such as: a) the rigid maintenance of plasma levels of 1,25(OH)_2_D_3 _at normal concentrations, even with low plasma concentrations of 25(OH)D (except in cases of chronic kidney disease and in the presence of high fibroblast growth factor-23 concentrations); b) plasma 25(OH)D levels are approximately 100-times greater than those of 1,25(OH)_2_D_3_; and c) hydroxylation of 25(OH)D to 1,25(OH)_2_D_3_ occurs in various tissues, thereby covering local needs [12].

In 2005, Hollis [13] considered that the optimal level of vitamin D would be that required to maintain parathyroid hormone (PTH) at appropriate levels. It is known that vitamin D deficiency leads to decreased serum calcium, which consequently stimulates the parathyroid glands to release PTH, thereby increasing renal reabsorption and bone calcium levels. In this regard, several studies have found a plateau of calcium absorption and adequate PTH levels, with 25(OH)D levels close to 30 ng/mL (75 nmol/L) [14,15,16,17,18]. However, the adequate levels of 25(OH)D for non-calcemic disorders has still not been established. 

### 2.1. Sources of Vitamin D

Factors such as latitude, season and time of day influence the cutaneous synthesis of vitamin D. During the summer, 7-dehydrocholesterol in the skin is more efficiently converted to previtamin D. Cutaneous synthesis of vitamin D is higher in low-latitude regions because of greater exposure to sunlight [19]. The use of sunscreen, the amount of melanin in the skin, types of clothing and high levels of air pollution may reduce skin exposure to UVB and result in decreased synthesis of vitamin D. Another important factor leading to hypovitaminosis D is changes in lifestyle, such as reduction of outdoor activities.

There are limited natural dietary sources of vitamin D. Not all countries have regulations requiring food fortification, and this leads to low consumption. Table 1 demonstrates the vitamin D content in selected foods. A recent review [20] showed the dietary requirements for adequate vitamin D nutritional status. For example, for 97.5% of the population aged 20-40 years, a mean intake during the winter of 8.7 µg/d would be needed in order to achieve 25(OH)D serum level greater than 25 nmol/L, and 41.1 µg/d for a 25(OH)D level of 80 nmol/L. The same dietary requirements have been observed among the elderly [21].

However, studies have demonstrated that several populations do not attain these dietary intake levels [22,23].

**Table 1:** Vitamin D_2_ and D_3_ content in selected foods, adapted from the USDA national nutrient database for standard reference, Release 22.

Foods (common portion sizes)	Vitamin D content (μg)	
	Vitamin D_2_	Vitamin D_3_
Salmon, cooked (155g)	0.0	36.1
Tuna, canned in oil (85g)	0.0	5.7
Sardines (24g)	0.0	4.8
Liver, beef cooked (85g)	0.0	1.0
Top sirloin, beef cooked broiled (85g)	0.0	0.2
Whole milk with vitamin D fortification (244g)	0.0	1.3
Whole milk, without vitamin D (244g)	0.0	0.1
Butter (5g)	0.0	1.5
Mushrooms, portabella, grilled(121g)	0.3	0.0
Mushrooms, portabella, grilled, exposed to UV light (121g)	13.1	1.0
Mushrooms, shiitake, cooked (72g)	0.7	0.1
Vegetables (kale, broccoli, spinach, tomato, carrots and lettuce) (100g)	0.0	0.0

## 3. Epidemiological and Observational Evidence

Cardiovascular diseases are the leading cause of death worldwide. According to the World Health Organization (WHO), these diseases affect 17.1 million people around the world and deaths occur predominantly in low and middle-income countries, almost equally in men and women. A reduction of 2 to 3 mmHg in systolic blood pressure is associated with a reduction of 10 to 15% in mortality from cardiovascular disease [24].

Recently, Giovannucci *et al.* [25] assessed the association between serum 25(OH)D and risk of coronary disease among men who participated in the Health Professionals Follow-up Study. Men with vitamin D deficiency (≤15 ng/ml or 37 nmol/L) were at significantly increased risk of developing myocardial infarction, compared with those with sufficient levels of vitamin D (≥30 ng/mL or 75 nmol/L) (RR 2.09; 95% CI: 1.24-3.54). 

Analyzing the population of NHANES III, Melamed *et al.* [26] observed that the prevalence ratio of peripheral arterial disease for the lowest 25(OH)D quartile, compared with the highest quartile (<44.5 and ≥73.8 nmol/L, respectively) was 1.80 (95% CI: 1.19-2.74). A similar situation has been observed in other studies [27,28] on the population of NHANES III, evaluating the associations between serum 25(OH)D and coronary heart disease, heart failure, stroke and peripheral arterial disease. 

Comparing vitamin D status among more than 3000 subjects, over a seven-year follow-up period, Pilz *et al*. [29] found that patients with severe vitamin D deficiency [25(OH)D < 25 nmol/L] had a risk of dying from heart failure or sudden cardiac death that was three to five times greater than among patients with optimal levels of vitamin D [25(OH)D ≥ 75 nmol/L]. In patients who had already had heart failure, low serum calcitriol concentrations were associated with critical end-stage outcomes [30]. 

Evaluating data from InCHIANTI, a prospective cohort study on aging, it was observed that participants who were in the lowest quartile of serum 25(OH)D (≤ 26.25 nmol/L) were at higher risk of all-cause mortality (HR 2.11, 95% CI: 1.22-3.64, *p *= 0.007) and mortality from cardiovascular disease (HR 2.64, 95% CI: 1.14-4.79, *p *= 0.02), compared with those in the highest quartile (≥66.25 nmol/L) [31].

Among adolescents [32], it was also demonstrated that low 25(OH)D levels were strongly inversely associated with cardiometabolic risk factors (systolic blood pressure and plasma glucose concentrations).

The involvement of vitamin D insufficiency with hypertension has also been demonstrated. Investigating the population over the age of 20 years who participated in NHANES III, Scragg *et al.* [3] found systolic and diastolic pressures that were respectively 3.0 and 1.6 mmHg lower in highest quintile of 25(OH)D (≥ 85.7 nmol/L), compared with the lowest quintile of vitamin D (25(OH)D ≤ 40 nmol/L). In the Nurses Health Study and the Healthy Professional Follow-up Study, the negative relationship between serum levels of vitamin D and hypertension was also demonstrated [33]. After four years of follow-up, the relative risk for men with low levels of serum 25(OH)D to develop hypertension was 6.13 (95% CI: 1.00-37.80), while for women it was 2.67 (95% CI: 1.05-6.97). After eight years of follow-up, the relative risk for men was 3.53 (95% CI: 1.02-12.3) and for women, 1.7 (95% CI: 0.92-3.16). 

## 4. Proposed Mechanisms for Vitamin D in Cardiovascular Disease

The mechanisms underlying the role of vitamin D in the prevention of heart disease remain incompletely explained. However, the mechanisms hypothesized involve the presence of VDR in various cells and its possible modulation of the expression of several genes. 1,25(OH)_2_D_3_ may interfere in the cascade of reactions and consequent functional capacity of certain cells. Such mechanisms include vitamin D as a negative regulator for renin and an inhibitor of cell proliferation and growth. 

### 4.1. Angiotensin-renin System

Inappropriate activation of the renin-angiotensin system may represent a major risk factor for hypertension and, consequently, for cardiovascular diseases. Several studies have indicated that serum levels of 1,25(OH)_2_D_3_ are inversely associated with blood pressure or plasma renin activity in normotensive and hypertensive subjects [34,35,36,37,38]. In a experimental study with wild-type mice, the research group of Yan Chun Li [7] showed that inhibition of 1,25(OH)_2_D_3_ synthesis led to an increase in renin expression, whereas 1,25(OH)_2_D_3_ injection led to renin suppression in the juxtaglomerular apparatus, independently of parathyroid hormone and calcium metabolism [39]. The same group [40] also demonstrated, in cell cultures, that 1,25(OH)_2_D_3_ directly suppressed renin gene transcription by means of a VDR-dependent mechanism. Elucidating this mechanism, a study found that 1,25(OH)_2_D_3 _suppressed renin gene expression in part by blocking the formation of the cyclic AMP response element [41]. These data suggest that vitamin D analogs and supplements may potentially be agents for controlling renin production and blood pressure.

Corroborating this hypothesis, Fryer *et al.* [42] evaluated the effects of paricalcitol and calcitriol on renin expression in C57/BL6 mice and showed that paricalcitol produces significant dose-dependent reductions in renin/GAPDH expression and calcitriol produced renin suppression. Additionally, Zhou *et al.* [43] demonstrated regulation of the renin-angiotensin system through supplementation of 1,25(OH)_2_D_3_ in 1-α hydroxylase knockout mice free of enzyme.

### 4.2. Role of vitamin D in cardiac tissue

Few *in vitro* and *in vivo* studies have evaluated the role of vitamin D in cardiac tissue. Carthy *et al.* [8], demonstrated *in vitro* that 1,25(OH)_2_D_3_ blocked the proliferation and growth of VSMC. In a recent study on administration of vitamin D analogs in cells cultures, Wu-Wong *et al.* [5] observed regulation of the expression of IGF1, Wilms tumor 1 and TGFß, which are three genes that are known to modulate cell proliferation. In addition, they observed downregulation of the expression of natriuretic peptide precursor B and thrombospondin 1, which inhibit cell proliferation. However, another study by the same group [44] suggested that elevated phosphorus affects VDR-mediated gene expression in human VSMC, and therefore the effect is not limited to VDR. 

Since VSMC is modulated by VDR, some studies have pointed towards its involvement in the endothelium. A study performed to evaluate endothelial function by brachial artery flow mediated dilatation in 23 asymptomatic vitamin D-deficient subjects found a positive correlation between endothelial function and 25(OH)D (r = 0.45; *p =* 0.001) [45]. 

With regard to the action of vitamin D on cardiomyocytes, a study [46] confirmed the presence of VDR, and that 1,25(OH)_2_D_3_ affected the growth, proliferation and morphology of murine cardiac myocytes (HL-1 cells) in cultures. The cells were treated with 1,25(OH)_2_D_3_, and increased expression of myotrophin with decreasing expression of atrial natriuretic peptide and c-myc were observed. Furthermore, the 1,25(OH)_2_D_3_ treatment also increased the expression and nuclear localization of the VDR in these cardiomyocytes. Another study [47] showed that 1,25(OH)_2_D_3_ treatment in a model of hypertensive rats subjected to a high-salt diet resulted in lower heart weight, myocardial collagen levels, left ventricular diameter and cardiac output, thus suggesting that it had an important preventive role in relation to the development of cardiac hypertrophy and consequent congestive heart failure. Corroborating these findings, it was found that paricalcitol supplementation in Dahl salt-sensitive rats that were also fed a high-salt diet attenuated the cardiac hypertrophy [48]. In Sprague-Dawley rats, maternal vitamin D deficiency led to increased left ventricle volume, greater cardiomyocyte numbers and size, and a higher proportion of mononucleated cardiomyocytes in the offspring at four weeks of age [49]. 

## 5. Vitamin D Supplementation Studies

In 2009, Zittermann *et al.* [50] conducted a double-blind, placebo-controlled trial in which 12 months of supplementation of 83.3µg of vitamin D was supplied to 200 women who had started a weight reduction program. They found that the group of supplemented women had greater decreases in PTH levels, triacylglycerides and tumor necrosis factor-α (TNF-α). Another important point was that weight loss did not differ between the vitamin D and placebo groups. Additionally, a study investigated whether vitamin D was associated with cytokine production [51]. It was found that vitamin D supplementation increased the anti-inflammatory cytokine production, such as IL-10, in patients with heart failure. 

A study on patients with predialysis chronic kidney disease showed that oral administration of alfacalcidol was associated with reduced risk of cardiovascular disease [52].

However, a randomized, double-blind, placebo-controlled trial on the population of the Women’s Health Initiative [53], which was administered 1,000 mg elemental calcium carbonate and 10µg of vitamin D_3_ daily, or placebo, found no reduction in mortality due to cardiovascular disease, but the hazard ratios trended in the direction of reduced risk. As an incidental finding, the daily amount of vitamin D_3_ in this study, like in other study using similar amounts of vitamin D supplementation [54], did not find any additional benefits. Furthermore, Bolland *et al.* [55] reported that calcium supplementation above the recommended levels in specific populations (elderly people or individuals with previous cardiovascular events) might increase the risk of cardiovascular events. 

In a double-blind, placebo-controlled study in 1987, Lind *et al.* [56] observed reductions in the blood pressure of 39 hypertensive patients with vitamin D supplementation. This reduction was also highlighted in another study on older women supplemented with calcium and vitamin D [57]. Another trial observed that administration of 1,25(OH)_2_D_3_ reduced blood pressure, as well as plasma renin activity and angiotensin II levels [58].

On the other hand, Thierry-Palmer *et al.* [59] increased the supply of vitamin D in the diet of salt-sensitive rats that were administered a high-salt diet and observed an increase in serum 25(OH)D, but their hypertension was not alleviated. These findings could suggest that there is a potential difference in the effects on the vitamin D endocrine system between salt-induced hypertension and essential hypertension.

Table 2 highlights the cardiovascular effects of vitamin D supplementation.

**Table 2:** Cardiovascular effects of vitamin D ssupplementation.

Study	Population	Supplementation type, dose and period	Main outcomes
Kimura *et al.*, 1999 [58]	Case report on a 42-year-old man.	Oral administration of 0.2 μg of calcitriol	↓ blood pressure, plasma renin activity and levels of angiotensin II.
Pfeifer *et al.*, 2001 [57]	148 women 70 years of age or older (74 patients in calcium group and 74 patients in vitamin-D-calcium group).	1,200 mg of CaCO_3_ or 1,200 mg of CaCO_3_+ 20 µg of cholecalciferol	In vitamin-D-calcium group, ↑ in 25(OH)D of 72% and ↓ in serum PTH of 17%, and in systolic blood pressure of 9.3%, heart rate ↓ 5.4%.
Schleithoff *et al.*, 2006 [51]	123 patients with congestive heart failure randomized into D(+) group and D(-) group	D(+) group received 50 µg of vitamin D_3 _+ 500 mg of Ca/d; D(-) group received placebo + 500 mg of Ca/d for 9 months	In D(+) group: ↓ PTH, ↑ IL-10 and TNFα remained constant. In D(-) group: ↑ TNFα. Survival rate did not differ significantly between groups.
Zittermann *et al.*, 2009 [50]	200 women who started a weight-loss program (100 patients in vitamin D group and 100 patients in placebo group).	83.3 μg/d of cholecalciferol for 12 months	↓PTH, triacylglycerides and TNF α.
Sigiura *et al.*, 2009 [52]	665 patients with predialysis chronic kidney disease (107 patients in alfacalcidol treatment group and 558 in non-treatment group).	0.25-0.5 μg/d of alfacalcidol for 24 weeks	Lower incidence of cumulative cardiovascular events in alfacalcidol treatment group.
LaCroix *et al.*, 2009 [53]	36,282 participants in *Women’s Health Initiative* (18,176 postmenopausal women in vitamin D group and 18,106 in placebo group).	1,000 mg CaCO_3_+ 10 µg/d of cholecalciferol for 7 years	No reduction in cardiovascular mortality.

## 6. Conclusion

Hypovitaminosis D has been observed worldwide and several studies have demonstrated a strong association between vitamin D status and cardiovascular diseases. There are few food sources of vitamin D, and the lack of food fortification in some countries, associated with low cutaneous synthesis, intensifies vitamin D insufficiency. Moreover, the understanding of the exact mechanisms through which 25(OH)D or the active form 1,25(OH)_2_D_3_ regulate the renin-angiotensin system and cell proliferation and growth (such as VSMC and endothelium cells) remains incomplete. In this regard, identifying correct relationships between vitamin D status and cardiovascular disease is an important matter that could contribute towards prevention of such diseases. In the meanwhile, health professionals should be aware of the potential negative implications of vitamin D insufficiency and make recommendations for their patients to improve their vitamin D status.
